# Magnesium Sulfate Enables Patient Immobilization during Moderate Block and Ameliorates the Pain and Analgesic Requirements in Spine Surgery, Which Can Not Be Achieved with Opioid-Only Protocol: A Randomized Double-Blind Placebo-Controlled Study

**DOI:** 10.3390/jcm10194289

**Published:** 2021-09-22

**Authors:** Hye-Min Sohn, Bo-Young Kim, Yu-Kyung Bae, Won-Seok Seo, Young-Tae Jeon

**Affiliations:** 1 Department of Anesthesiology and Pain Medicine, Ajou University School of Medicine, Ajou University Hospital, Suwon 16499, Korea; wonseok612@aumc.ac.kr; 2 Department of Anesthesiology and Pain Medicine, Seoul National University Bundang Hospital, Seong-nam 13620, Korea; to123215@gmail.com (B.-Y.K.); vansuri27@gmail.com (Y.-K.B.); anesthesiology@nate.com (Y.-T.J.)

**Keywords:** intraoperative neurophysiological monitoring, magnesium sulfate, neuromuscular blocking drugs, opioid consumption, postoperative pain, spine surgery

## Abstract

Spine surgery is painful despite the balanced techniques including intraoperative and postoperative opioids use. We investigated the effect of intraoperative magnesium sulfate (MgSO_4_) on acute pain intensity, analgesic consumption and intraoperative neurophysiological monitoring (IOM) during spine surgery. Seventy-two patients were randomly allocated to two groups: the Mg group or the control group. The pain intensity was significantly alleviated in the Mg group at 24 h (3.2 ± 1.7 vs. 4.4 ± 1.8, *p* = 0.009) and 48 h (3.0 ± 1.2 vs. 3.8 ± 1.6, *p* = 0.018) after surgery compared to the control group. Total opioid consumption was reduced by 30% in the Mg group during the same period (*p* = 0.024 and 0.038, respectively). Patients in the Mg group required less additional doses of rocuronium (0 vs. 6 doses, *p* = 0.025). Adequate IOM recordings were successfully obtained for all patients, and abnormal IOM results denoting warning criteria (amplitude decrement >50%) were similar. Total intravenous anesthesia with MgSO_4_ combined with opioid-based conventional pain control enables intraoperative patient immobilization without the need for additional neuromuscular blocking drugs and reduces pain intensity and analgesic requirements for 48 h after spine surgery, which is not achieved with only opioid-based protocol.

## 1. Introduction

Patients may experience significant pain after major spine surgery despite the balanced techniques including intraoperative opioids and postoperative patient-controlled analgesia (PCA) [1]. Studies have reported that 20% of patients who undergo spine surgery complain of only a slight improvement in pain intensity despite conventional treatment [1,2], and many develop chronic pain after surgery. Effectively controlled postoperative pain helps patients recover faster, lessens excessive analgesic consumption, and alleviate serious complications and poor prognoses [3].

Magnesium is an abundant cation in the body and essential to numerous physiological activities. Magnesium sulfate (MgSO_4_) as an adjuvant to general anesthesia is related to multiple mechanisms of action, including lessening central excitability via *N*-methyl-d-aspartate (NMDA) receptor antagonism, delaying the development of opioids tolerance, and effects on balance of neurotransmitters [4,5].

Total intravenous anesthesia (TIVA) is widely used in spine surgery these days, as intraoperative neurophysiological monitoring (IOM) has aided spine surgery with the measured evoked potentials provide much information on the integrity of the sensory and motor neurone pathways during the surgery [6,7]. During IOM, the additional use of rocuronium after anesthesia induction is generally limited or avoided to ensure the accurate measurement of somatosensory evoked potentials (SSEPs) and motor evoked potentials (MEPs); however, anesthesiologists fear that such anesthetic restrictions might lead to the patient’s unacceptable movement during the procedure or the shallow depth of anesthesia. As such, when the use of neuromuscular blocking (NMB) drugs needs to be restricted, MgSO_4_ administration can enhance the potency or duration of neuromuscular blockade, thereby reducing the need for NMB drugs during surgery [7]. At the same time, it should be considered that MgSO_4_ during IOM could interfere with the precise assessment of significant changes in SSEPs and MEPs [8]. Nevertheless, no clinical trial has evaluated the additional efficacy and applicability of MgSO_4_ when used with opioids, especially under IOM during spine surgery to date.

We hypothesized that the intraoperative administration of MgSO_4_ could reduce pain intensity and total opioid consumption after spine surgery compared to conventional opioids-based therapy. In addition, we investigated the acceptable safety profile of MgSO_4_ during IOM with regard to concomitant use of NMB drugs, as well as preventive MgSO_4_ as an essential element of multimodal analgesia through this prospective, randomized, double-blind study.

## 2. Materials and Methods

### 2.1. Study

This study was approved by the Institutional Review Board of Seoul National University Bundang Hospital (IRB No. B-1804-462-002), Seong-nam, Korea (Chairperson Prof Jae-Ho Lee) on 2 July 2018 and registered at cris.nih.go.kr (accessed on 9 August 2018 registration number KCT0004173). All patients provided informed consent.

### 2.2. Patients

All patients provided informed consent. A total of 72 patients aged 20–79 years were enrolled in this prospective randomized study if they receive IOM while undergoing major spine surgery; decompression, fusion, laminoplasty, or tumorectomy. Patients were excluded from the study for the following reasons: they had an American Society of Anesthesiologists’ (ASA) physical status classification of IV or greater, body mass index < 15 or >35 kg m^−2^, were not using postoperative intravenous PCA, presence of renal, hepatic, or cardiovascular dysfunction, neuromuscular disease, admission to the intensive care unit at the end of surgery, under medication with calcium channel blockers or magnesium, refusal to participate in the study, anesthesia time more than 5 h preventing excessive infusion of MgSO_4_, any other physical or mental illness rendering them not capable of answering the pain score.

### 2.3. Anesthesia and Intervention

Before surgery, patients were asked to rate their pain using a numeric rating scale (NRS) from 0 (free of pain) to 10 (worst pain imaginable) by a member of the research team. Anesthesia was induced by continuous infusion of propofol with remifentanil through a target-controlled infusion pump (Orchestra^®^, Fresenius vial, France). Rocuronium 0.6 mg kg^−1^ was administered to facilitate tracheal intubation. Following intubation, patients received either MgSO_4_ or saline according to a randomization list: the Mg group (MgSO_4_ 30 mg kg^−1^ i.v. for 10 min, followed by continuous infusion of 15 mg kg^−1^ h^−1^ during the surgery) or the control group (same volume of isotonic saline) by the attending anesthesiologists. The effect-site concentration of propofol (3–6 µg ml^−1^) was set to ensure a bispectral index (BIS) (A-2000 BIS™ monitor; Aspect^®^ Medical Systems Inc., Norwood, MA, USA) between 40 and 55. The target concentration of remifentanil (1–5 ng mL^−1^) was tuned to maintain arterial pressure and heart rate within 20% of the preoperative values, but not below 1 ng mL^−1^. Nitrous oxide was not allowed because of its NMDA antagonistic properties. Controlled ventilation was adjusted to an end-tidal CO_2_ of 4.0–4.7 kPa. The train-of-four (TOF) Watch SX^®^ (Organon Ltd., Dublin, Ireland) was stimulated on the ulnar nerve near the wrist at 15 min intervals. The target was at least ≥ 2-count response of TOF stimulation before starting baseline MEP monitoring.

Patients were allowed to receive top-up dose of 10 mg rocuronium whenever spontaneous respiration or movement interfered with the surgery or when the surgeon requested. The muscles recorded for TOF and IOM were kept warm with a warm blanket to maintain optimal body temperature. Anemia, hypotension and hypoxemia were avoided. Partial neuromuscular blockade was not considered. After the main procedure, MgSO_4_ or saline was discontinued and IOM was also terminated. The patients were extubated following of 0.01 mg kg^−1^ glycopyrrolate and 0.03 mg kg^−1^ neostigmine administration and assurance of TOF ratio ≥0.90, then transferred to the postanesthetic care unit (PACU).

Thirty minutes before the end of surgery, to control initial postoperative pain, 1000–2000 µg fentanyl was used in all patients depending on age and weight in intravenous PCA pumps (total volume of 100 mL, bolus of 1 mL, and lockout interval of 10 min without basal infusion). For postoperative nausea and vomiting (PONV) prevention, prophylactic palonosetron—a second-generation 5-HT_3_ receptor antagonist (0.075 mg, intravenous)—was used prior to starting PCA infusion, and preoperative dexamethasone (10 mg) was administered equally in both groups. Metoclopramide 10 mg, and ramosetron 0.3 mg were used as rescue antiemetics at least 6 h interval postoperatively.

All patients were given the same anesthetics and multimodal pain protocol. Preoperative analgesics were administered as needed on the ward with acetaminophen 650 mg, meperidine 25 mg, or fentanyl 50 mcg. Intraoperatively, remifentanil was infused, and PCA was started. For postoperative acute pain management, if necessary, rescue analgesics (morphine 5 mg, propacetamol 1 g, meperidine 25 mg, fentanyl 50 mcg, acetaminophen 1 g premix, ketorolac 30 mg, nefopam 20 mg) were administered in the recovery room and in the wards. Once the patients resumed oral intake, acetaminophen 1300 mg and oxycodone 5 mg were administered every 6 h.

### 2.4. Assessment of Outcomes

The cumulative fentanyl consumption of PCA solution was recorded at 6, 24, and 48 h after surgery by the blinded investigator. Consumption of rescue analgesics and postoperative pain was also evaluated using an NRS at the same time period. The total amount of rocuronium and PONV, rescue antiemetics, and neurophysiological event logs were recorded. Computer-generated block randomization with blocks of size 4 was used and group assignment was kept in sealed envelopes. The preparation and administration of the parenteral drug as well as the collection and measurement of data were performed by doctors and nurses who were blinded to the study group allocations.

### 2.5. Intraoperative Neurophysiological Monitoring

After anesthesia induction, IOM was started using a Xltek Protektor IOM (Optima Medical Ltd., London, UK). The stimulating and recording electrodes were placed and baseline potential of MEP and SSEP waveforms were obtained, before initiation of the surgical procedures. According to the International 10–20 electroencephalogram system, needle-type electrodes were placed subcutaneously over the motor cortex region at C3 and C4 for left and right hemispheric stimulation [9,10]. Bilateral MEPs were recorded at the adductor hallucis, tibialis anterior, and several muscles in extremities were added depending on the surgical area [11]. Somatosensory EP responses from both the ulnar and posterior tibial nerves were monitored. Free-running electromyography (EMG) was recorded in the specific innervated muscles, when applicable. Once the baseline signal was obtained, any change in stimulation intensity was documented, and the baseline waveform was compared to the waveforms obtained afterward. Warning criteria for notification was defined as a decrease of more than 50% of MEP of SSEP amplitude, or a loss of MEP for 3 consecutive trials [11]. When the acquisition of baseline MEP monitoring before starting the surgery or the ongoing MEP during the surgery was not proceeding properly, the neurologist consulted the anesthesiologist on whether to administer the reversal agent in advance. If TOF count was ≥2-count response at the time of request, the anesthesiologist considered reverse injection, but it was at the discretion of the anesthesiologist in charge of the operating room.

### 2.6. Sample Size Calculation

The sample size was determined based on the result of the previous investigation [5]. The consumption of morphine equivalent at postoperative 24 h was 44.7 (16.6) mg for the control group, and a decrease of 25% in opioid consumption was considered to be clinically significant. A power analysis indicated that 36 patients should be recruited for each group for a power of 80% and error of 0.05.

### 2.7. Statistical Analysis

Measured continuous variables are shown as absolute values, means (standard deviation), percentages, or median (interquartile range). Continuous variables were compared with Student’s t-test or the Mann–Whitney *U* test. Incidence data were compared using the *X*^2^ test or Fisher’s exact test according to the expected counts. All statistical analyses were performed using SPSS software (Version 25.0; IBM SPSS Inc., Armonk, NY, USA), and statistical significance was defined as *p*-value < 0.05 (2-sided).

## 3. Results

**Figure 1:**
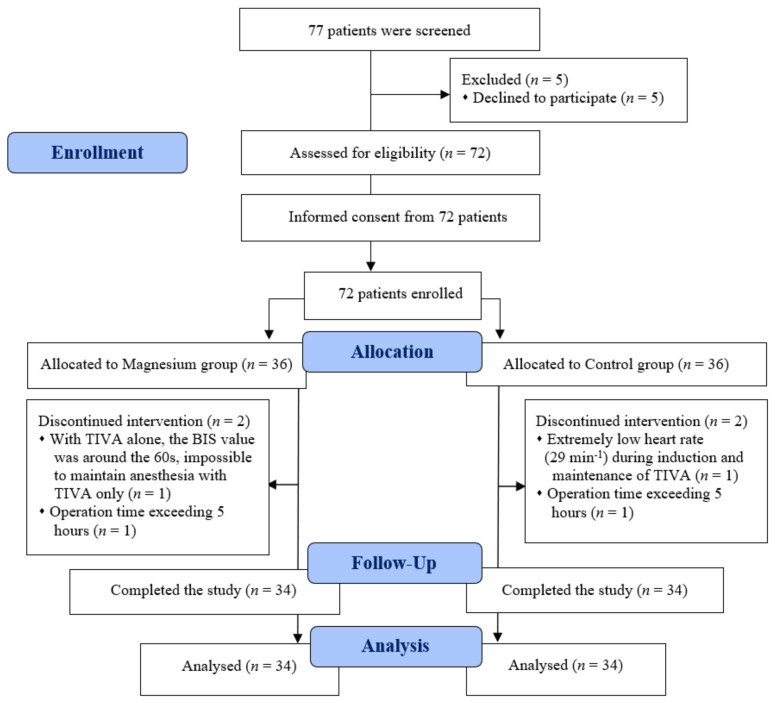
CONSORT diagram. BIS, bispectral index; TIVA, total intravenous anesthesia.

Among the 72 patients assessed for eligibility, two in each group were excluded from analyses. One patient from each group had an operation time exceeding 5 h; in another patient from the Mg group, maintaining anesthesia via TIVA alone was impossible, because the BIS value remained around the 60 s irrespective of the increase in the TIVA dose; another patient from the control group showed an extremely low heart rate (29 min^−1^) during maintenance with TIVA (Figure 1). The remaining 68 patients completed the study and were finally included in the analyses. Table 1 presents the patient characteristics and surgical factors. No differences were observed between the two groups.

Regarding the primary outcome measure, pain intensity was lower in the Mg group at 24 h (3.2 ± 1.7 vs. 4.4 ± 1.8, *p* = 0.009) and 48 h (3.0 ± 1.2 vs. 3.8 ± 1.6, *p* = 0.018) after surgery than in the control group (Table 2). Patients in the Mg group consumed 33.1% (*p* = 0.024) and 32.3% (*p* = 0.038) less fentanyl via PCA on average during the 24 and 48 h after surgery (Table 2).

Rocuronium was significantly less used as top-up doses in Mg group (0 vs. 6 cases, *p* = 0.025). Adequate IOM recordings were obtained for all patients. Abnormal IOM results denoting warning criteria (MEP amplitude decrease >50%, SSEP amplitude decrease > 50%) were similar (1 vs. 5, *p* = 0.197) and most of them recovered later (Table 3). Early administration of reversal agents before or during the main procedure for accurate acquisition of MEP or SSEP recordings was similar (five cases in the Mg group vs. two in the control group) (Table 4). There were no differences between groups in terms of rescue analgesics, PONV incidence, and rescue antiemetics until 48 h after the surgery (Table 4 and Table 5).

All the patients met the PACU discharge criteria within an hour of the surgery, and Aldrete score was similar. None of the patients showed any prolonged sedation, delayed recovery, respiratory depression, or electrocardiographic changes. No compatibility issues related to MgSO_4_ infusion with IOM, such as newly occurred postoperative motor deficit without MEP changes, were observed. Intraoperative ionized Mg levels (*p* < 0.001) were higher in the Mg group.

## 4. Discussion

We demonstrated that intraoperative preventive MgSO_4_ combined with fentanyl-based multimodal protocol reduces postoperative pain intensity and opioids consumption in patients undergoing major spine surgery with IOM, with maximal effects at 24 and 48 h postoperatively. Intraoperatively, TIVA with MgSO_4_ administration provided patient immobilization and adequate IOM without the need for additional NMB drugs.

Pain intensity was significantly lower in the Mg group than in the control group at 24 and 48 h after surgery. Although the main component of preoperative pain is essentially different from postoperative pain, the Mg group showed a significant reduction in postoperative pain (4.7 vs. 3, *p* < 0.05), while the pain in the control group remained almost the same as the preoperative level of 4.4 at 24 h after surgery. As with our results, conventional opioid-based pain protocol in surgical patients often remain in suboptimal pain control [12]. Spine surgery is a painful procedure; therefore, balanced general anesthesia is widely used [13] and the current practice of the most common perioperative strategy also relies almost exclusively on opioids [1,14]. Severe pain and associated large amounts of opioids consumption has undesirable side effects; increases morbidity, interferes postoperative exercise, delays rehabilitation and functional outcomes [15]. Often, patients undergoing major spine surgery are already suffering from long-lasting pain before surgery, making it more difficult to manage any additional acute pain after surgery [16].

Recognition has progressed in multimodal pain management, which combines both opioids and non-opioid adjuvants to maximize pain relief through synergistic effect, and lower analgesic doses and minimize potential complications [13,14]. Nociception is the propagation of potentially harmful and noxious stimuli through the sensory system. Nociceptive stimuli activate the release of glutamate which acts on NMDA receptors, promoting the entry of calcium into the cell and triggering central sensitization [17]. As an NMDA-receptor antagonist, magnesium not only lessens the generation of noxious stimuli, but also decreases neuronal sensitivity to the opioid receptor [18]. Therefore, when administered together with an opioid, magnesium prevents tolerance to opioid analgesia [17] and leads to lesser opioid consumption while achieving analgesia even after the termination of magnesium administration [4], with less untoward side-effects.

Preventive MgSO_4_ reduced total opioid consumption up to 48 h after surgery in the current study, which is consistent with prior reports but of greater magnitude (33%) [19]. In particular, due to this opioid-sparing effect, MgSO_4_ has more recently been proposed as one component in different multimodal analgesic concepts in the context of opioid-free anesthesia [20]. We showed clinical utility in combination with MgSO_4_, which opioids alone cannot, to manage intraoperative nociception and postoperative pain. Magnesium, which has been proven for over three decades, can be a good, easy and inexpensive option, although not yet widely used, owing to its opioid-sparing and analgesia-potentiating effects. In a recent review paper, MgSO_4_ was also capable of reducing anesthetic requirements, which is consistent with many other previous studies [19]. Unfortunately, we did not get the total amount of TIVA in our study, but when the initial bolus of MgSO_4_ was between 30 and 50 mg kg^−1^, followed by continuous infusion of 8–20 mg kg^−1^, the meta-analysis yielded a result of −213 mg less for maintenance propofol, −112 mcg for remifentanil, which were favorable for the Mg group.

The reason why the demand for top-up of rocuronium was lower in the Mg group (0 cases vs. 6 in control group) is because there is an advantages in the simultaneous use of rocuronium and MgSO_4_; rocuronium is a competitive antagonist to acetylcholine, and magnesium-induced acetylcholine reduction from the presynaptic nerve terminal has the possibility of increasing the neuromuscular potency of rocuronium [21]. The myorelaxant-sparing effect of MgSO_4_ made it possible to withhold further use of NMB drugs to ensure adequate depth of anesthesia. If the use of rocuronium should be minimized, MgSO_4_ may help reduce patient movement due to its muscle-relaxing effects. There were no cases where the apparent movement of the patient made the surgeon uncomfortable or jeopardized the patient’s safety during the surgery. MgSO_4_ has been reported to increase both the duration of intense neuromuscular blockade and the period of no response to nerve stimulation [22].

We administered only 60% of the MgSO_4_ bolus used in previous studies [5,23,24] to avoid interference with the IOM, since IOM can be a very critical finding in some patients, we did not want to obscure it. Additionally, as there was no existing literature on what dose of MgSO_4_ is safe or feasible during IOM we decided to administer a conservative dose, which is known to be effective (30–50 mg kg^−1^) in pain control. Unfortunately, the maximal magnesium levels were not measured in this study. Following the administration of the 4 g loading dose (vs. an average of 1.8 g in the current study), the serum magnesium levels rose sharply to double the baseline levels and achieved peak serum concentration (1.48–1.70 mmol L^−1^) at about 0.5 h; thereafter, the levels remained within a similar range (steady-state level) until 24 h [24,25]. Since the current intraoperative test was withdrawn while the bolus was entering, the ionized magnesium level rose only moderately. Measuring the magnesium level after injection of all the magnesium would have shown more marked concentration differences.

We rigorously tested the accurate acquisition of IOM and its full role in the midst of continuous infusion of MgSO_4_. MEP, SSEP, and EMG monitoring was completed in all patients (Table 3). We observed IOM and TOF count for this changing situation, and subsequent IOM and surgery were completed without any specific disturbance or interference. Only the related muscles or limb was affected in some cases, and there was no case where the entire IOM was affected, making examination impossible or requiring discontinuation of MgSO_4_ infusion. In another unusual case report, the amplitude of MEP was lost overall due to the large dose of magnesium as a bolus administration during IOM; then the responses returned to near baseline about 20 min later, without any new postoperative deficits thereafter [26]. When the magnesium bolus is suddenly administered in the middle of the IOM process, the abrupt occurrence of MEP changes should be considered [8].

There is a fundamental difference in design between our research and previous ones using MgSO_4_ in that the neuromuscular blockade of the current study should be relatively weakened at the beginning of surgery [5,22,27]. IOM baseline signal acquisition was started between 30 min and 1 h after the initiation of the magnesium loading dose [25], which seemed to coincide with the time point when the magnesium concentration was at its peak. Moreover, as magnesium was continuously infused, the concentration of accumulated magnesium would have gradually increased. In previous studies, magnesium helped deepen anesthesia at this point, since this was usually just before skin incision [5,24] that sensitizes of stimulated nociceptors [17]. Conversely, we had to unstring neuromuscular blockade by the start of surgery, with at least two twitches of the TOF detectable. At the request of a neurologist and surgeon, the reversal agents were administered early (five in the Mg group and two in the control group) before and during surgery if necessary, despite the fact that the depth of block was already at moderate or shallower for the following reasons. Neuromuscular reversal and the process until full recovery can have a variety of responses, in terms of the time it takes to reach shallow or minimal block depending on the type of NMB drugs, type of reversal agents, doses (full doses vs. lower doses), and degree of block at the time of reversal injection [28]. Furthermore, anticholinesterases have an upper limit to the depth of block that they can antagonize thoroughly, even at these relatively advanced levels of neuromuscular recovery.

Our findings led us to propose better study design modifications in the future. First, the initiation and termination of the magnesium injection should be as early as possible for the pretreatment period not to overlap with the critical acquisition point of the IOM; albeit only slightly, to undermine the influence of magnesium bolus to IOM [22,27]. Second, it is advisable to measure the correct TOF ratio with strict quantitative neuromuscular monitoring at the request of reversal injection. Third, it is necessary to review with the surgeon and neurologist whether the specific intensity of NMB and specific timing necessarily requires reverse injection, or, conversely whether it is sufficiently antagonized As magnesium can also exert an effect of enhancing residual paralysis, it should be noted that the recovery of TOF ratio of 0.9 or higher does not guarantee protection against magnesium-induced recurrent NMB [21].

The incidence of PONV was also not different (29.4% vs. 23.5%) between the groups, despite differences in opioid usage. The low incidence in both groups may have benefitted from the appropriate stringent antiemetic strategy used in our institution. The TOF ratio before extubation was greater than 0.9 in all patients. No delayed emergence from anesthesia, hypoxemia, or critical respiratory events were reported. There were no differences in intraoperative hypotension, hypothermia, the use of phenylephrine, ephedrine and atropine.

The present study has several limitations. First, several types of surgery were included in this study, therefore, different pain-related outcomes may have had an impact on the main results. Especially, in relation with spinal surgical level, lumbar surgery is known to be quite different regarding pain from the cervical surgery. In our subgroup analysis, fentanyl consumption and pain scores were lower in cervical surgeries at postoperative 24 and 48 h than non-cervical surgeries. Second, the infused doses of propofol or remifentanil were not collected. Therefore, we could not observe/prove the anesthetic-sparing benefit of MgSO_4_, reducing the requirements of TIVA—which has often been demonstrated in previous studies [5,19,23]. Constant concentrations are usually maintained because rapid alterations of anesthetics during IOM make interpretation of evoked potentials difficult, resulting in false positive/negative results. Third, details of SSEP and MEP, i.e., average amplitudes were not collected from a technical standpoint, and these factors vary across institutions, potentially limiting the generalization of our IOM finding. Fourth, MgSO_4_ was used as essentially one addition to intraoperative opiates. Many multimodal analgesic regimens employ multiple drugs and presents a dizzying number of permutations and combinations to potentially study. Whether or not magnesium interacts with other typically used drugs for multimodal analgesia remains open to question. Lastly, long-term follow-up of pain control was not performed. As the types of surgeries were heterogeneous, a simple comparison of follow-up NRS between the groups would have been futile.

## 5. Conclusions

In conclusion, preventive MgSO_4_ reduces postoperative pain intensity and opioids consumption at 24 and 48 h after surgery. The intraoperative benefit of MgSO_4_ is the lack of any interference to neurophysiological monitoring without the need for additional NMB drugs while maintaining depth of block. With these reasons, we recommend that anesthesiologists and surgeons consider MgSO_4_ as an essential part of multimodal analgesic therapy to improve patient safety and outcomes for the patients who are undergoing painful surgery.

## Figures and Tables

**Table 1:** Baseline characteristics of patients and perioperative data assigned to magnesium or the control group.

	Magnesium Group (*n* = 34)	Control Group (*n* = 34)	*p*-Value
Gender (male/female)	14/20	17/17	0.465
Age (year)	56.5 ± 13.7	56.5 ± 14.7	0.993
Height (cm)	160.8 ± 10.8	161.9 ± 8.7	0.650
Weight (kg)	60.8 ± 13.7	63.7 ± 12.7	0.375
ASA physical status score (I/II/III)	7/22/5	7/22/5	1.000
Diagnosis, *n* (%)			
Herniated disc	7 (20.6%)	6 (17.6%)	0.758
Myelopathy	6 (17.6%)	6 (17.6%)	1.000
Tumor	11 (32.4%)	12 (35.3%)	0.798
Ossification of ligament	2 (5.9%)	4 (11.8%)	0.673
Spinal stenosis	3 (8.8%)	2 (5.9%)	1.000
Other (fracture, deformities)	5 (14.7%)	4 (11.8%)	1.000
Number of spines operated on			
Levels	2 (1–3)	1.5 (1–2)	
1 level, *n* (%)	17 (50%)	14 (41.2%)	0.465
≥2 level, *n* (%)	17 (50%)	20 (58.8%)	0.465
Surgical Level			
Cervical (anterior/posterior approach)	20 (58.8%) (6/14)	18 (52.9%) (7/11)	0.625
Thoracic	5 (14.7%)	12 (35.3%)	0.05
Lumbar	4 (11.8%)	2 (5.9%)	0.673
Cervicothoracic	2 (5.9%)	0	0.493
Thoracolumbar	3 (8.8%)	2 (5.9%)	1.000
Type of Surgery			
Decompression/laminectomy	4 (11.8%)	6 (17.6%)	0.493
Laminoplasty/duroplasty	8 (23.5%)	4 (11.8%)	0.203
Fusion	10 (29.4%)	12 (35.3%)	0.604
Tumorectomy	11 (32.4%)	12 (35.3%)	0.798
Corpectomy	1 (2.9%)	0	1.000
Duration of surgery (min)	144.1 ± 52.0	158.5 ± 42.7	0.216
Duration of anesthesia (min)	204.9 ± 51.0	220.9 ± 44.9	0.173

Values are number (proportion), mean ± standard deviation or median (interquartile range). ASA, American Society of Anesthesiologists.

**Table 2:** Cumulative fentanyl consumption and pain scores during the first 48 h after surgery.

	Magnesium Group (*n* = 34)	Control Group (*n* = 34)	*p*-Value
Fentanyl consumption (mcg)			
Postoperative 6 h	104.3 ± 88.3	115.2 ± 79.9	0.595
Postoperative 24 h	284.8 ± 234.7 *	426.2 ± 268.4	0.024
Postoperative 48 h	503.2 ± 436.4 *	743.4 ± 496.9	0.038
Pain scores (NRS)			
Pre-operative period	4.7 ± 2.5	4.4 ± 2.5	0.632
PACU	4.8 ± 2.5	5.0 ± 2.3	0.801
Postoperative 2 h	4.3 ± 2.5	4.1 ± 2.2	0.716
Postoperative 6 h	4.1 ± 1.4	4.4 ± 1.6	0.341
Postoperative 24 h	3.2 ± 1.7 *^,†^	4.4 ± 1.8	0.009
Postoperative 48 h	3.0 ± 1.2 *^,†^	3.8 ± 1.6	0.018

Values are mean ± standard deviation. NRS, numeric rating scale; PACU, postanesthetic care unit. * *p* < 0.05 between groups. ^†^ *p* < 0.05 from preoperative value in the magnesium group.

**Table 3:** Intraoperative neurophysiological monitoring results.

	Magnesium Group (*n* = 34)	Recovery	Control Group (*n* = 34)	Recovery	*p*-Value
MEP change	1 (2.9%)	Only the affected limb decreased and the MEP recovered 10 min later	5 (14.7%)	3 recovered 2 completed operation with a reduced MEP response (>80%) of specific operation-related muscles	0.197
SSEP change	0 (0%)		1 (2.9%)	Recovered 5 min later	1.000
EMG change	7 (20.6%)	No correlation to post-op outcomes	7 (20.6%)	No correlation to post-operation outcomes	1.000
Warning criteria (MEP + SSEP) occurrence	1 (2.9%)		5 (14.7%) *		0.197

Values are number (proportion). EMG, electromyography; MEP, motor evoked potentials; SSEP, somatosensory evoked potentials. * After subtracting 1 overlapping subject.

**Table 4:** Intraoperative anesthesia related variables.

	Magnesium Group (*n* = 34)	Control Group (*n* = 34)	*p*-Value
Crystalloids (mL)	1163.2 ± 424.1	1171.6 ± 355.5	0.930
Colloids (mL) intraoperatively	170.6 ± 295.2	163.2 ± 315.1	0.921
Estimated blood loss (mL)	225.0 ± 208.6	195.9 ± 209.1	0.567
Packed red blood cells, *n* (%)	0	1 (2.9%)	1.000
Transfusion via cell-saver, *n* (mL)	1 (100)	0	1.000
Rocuronium induction (mg)	44.6 ± 9.7	47.4 ± 8.9	0.143
Rocuronium added dose, *n* (%)	0	6 (17.6%)	0.025
Timing of reversal agents injection			
During IOM baseline acquisition before surgery	1 (2.9%)	2 (5.9%)	1.000
Within 1 h after the skin incision	4 (11.8%)	0 (0%)	0.114
At the end of surgery	29 (85.3%)	32 (94.1%)	0.427
Ionized Mg (Mg^++^) (mmol L^−^^1^)	0.71 ± 0.15	0.55 ± 0.11	0.000

Values are number (proportion) or mean ± standard deviation. IOM, intraoperative neurophysiological monitoring.

**Table 5:** Incidence of postoperative nausea and vomiting, rescue antiemetics, and analgesics during the first 48 h after surgery.

	Magnesium Group (*n* = 34)	Control Group (*n* = 34)	*p*-Value
PONV overall	10 (29.4%)	8 (23.5%)	0.549
PONV at 6 h	2 (5.9%)	3 (8.8%)	0.642
PONV at 24 h	6 (17.6%)	8 (23.5%)	0.549
PONV at 48 h	4 (11.8%)	4 (11.8%)	1.000
Rescue antiemetics	4 (11.8%)	4 (11.8%)	1.000
Rescue opioids at postop 6 h	6 (17.6%)	9 (26.5%)	0.111
Rescue opioids at postop 24 h	4 (11.8%)	4 (11.8%)	1.000
Rescue opioids at postop 48 h	1 (2.9%)	1 (2.9%)	1.000
Rescue NSAIDs overall	7 (20.6%)	4 (11.8%)	0.323

Values are number (proportion). NSAIDs, non-steroidal anti-inflammatory drugs; PONV, postoperative nausea and vomiting.

## Data Availability

The data presented in this study are available from the corresponding author on reasonable request.
